# Frequency and Manifestations of Autoimmunity Among Children Registered in the Kuwait National Primary Immunodeficiency Registry

**DOI:** 10.3389/fimmu.2020.01119

**Published:** 2020-06-02

**Authors:** Michel J. Massaad, Mohammad Zainal, Waleed Al-Herz

**Affiliations:** ^1^Department of Experimental Pathology, Immunology and Microbiology, Faculty of Medicine, American University of Beirut, Beirut, Lebanon; ^2^Department of Pediatrics and Adolescent Medicine, American University of Beirut Medical Center, Beirut, Lebanon; ^3^Department of Quantitative Methods and Information Systems, College of Business Administration, Kuwait University, Kuwait City, Kuwait; ^4^Department of Pediatrics, Faculty of Medicine, Kuwait University, Kuwait City, Kuwait; ^5^Allergy and Clinical Immunology Unit, Pediatric Department, Al-Sabah Hospital, Kuwait City, Kuwait

**Keywords:** primary immunodeficiencies, autoimmunity, immune dysregulation, registry, epidemiology, manifestations, mortality

## Abstract

**Objectives:** To present a prospective report on the characteristics of autoimmune manifestations in patients with primary immunodeficient children registered in the Kuwait National PIDs Registry (KNPIDR).

**Methods:** The data were obtained from the Kuwait National Primary Immunodeficiency Disorders Registry during the period of January 2004 to December 2019.

**Results:** A total of 286 PID children were registered in KNPIDR during the study period with a predominance of immunodeficiencies affecting cellular and humoral immunity followed by combined immunodeficiencies with associated syndromic features and diseases of immune dysregulation. Fifty-seven (19.9%) patients presented with a total of 107 autoimmune manifestations. There was no significant statistical association between autoimmune manifestations and gender. Patients with autoimmune manifestations were older at onset of PID symptoms compared to those with no such manifestations, but this did not reach level of significance. The diagnosis delay was longer in patients with autoimmune manifestations compared to those with no such manifestations (*p* = 0.038). Forty-seven percent of these manifestations were among the presenting symptoms while 53% were documented later during the course of the disease. Fifty-seven percent of the patients developed 1 autoimmune manifestation, 30% developed 2 such manifestations, and 16% had ≥3 autoimmune manifestations. The most common autoimmune manifestation was cytopenia, followed by gastrointestinal manifestations and manifestations of the skin, hair, and nails. Autoimmune cytopenia were more common in patients with immune dysregulation syndromes, while gastrointestinal and skin manifestations predominate in patients with immunodeficiencies affecting cellular and humoral immunity and endocrine manifestations were more common in immune dysregulation syndromes. There were significant statistical associations between developing autoimmune manifestations and death as well as PID categories, being more common in patients with immune dysregulation. The frequency of autoimmunity was high among patients with RAG, WAS, STAT5b, NF-κB2, Fas, FasL, LRBA, APECED, IL-10, and C4 deficiencies.

**Conclusions:** Autoimmunity is frequent in patients with PIDs in Kuwait. This should prompt the suspicion of a PID in patients who present initially with autoimmunity, especially autoimmune cytopenia. Such patients should be managed with extra care since they are at a higher risk of death.

## Introduction

Primary immunodeficiency diseases (PIDs), or inborn errors of immunity, are a heterogeneous group of genetic disorders that impair the development and/or function of immune cells leading to defective and/or uncontrolled immune responses. A defect in at least one of more than 400 genes have been associated with the development of PIDs, resulting in various combinations of recurrent infections, autoimmunity, lymphoproliferation, granulomatous process, atopy, and malignancy (1).

Although infections are the most common manifestation of PIDs, autoimmunity, and inflammation are frequent and are sometimes the first to manifest (2–5). In a retrospective review of the French National Primary Immunodeficiencies Registry, it was found that autoimmune or inflammatory symptoms developed in 26.2% of patients with PIDs throughout their lifetime (6). It is now recognized that the autoimmunity observed in some monogenic disorders traditionally associated with PIDs, is due to the same gene defect that resulted in the PIDs, as is the case in diseases due to exaggerated type I interferon production, defective complement function, defective T and B cell receptor rearrangement, homeostasis, and negative selection, and defective regulatory T cell (Treg) development or function (7). Furthermore, a genome-wide association study done on a large number of patients with systemic lupus, traditionally believed to be a polygenic disorder (8), identified several genes associated with increased risk of developing the disease, 15 of which were identical to those previously associated with PIDs (9). These data provided a strong indication of a common etiology for some PIDs and autoimmune or inflammatory diseases.

PIDs are considered rare diseases from a global perspective, however, they are underestimated worldwide (10, 11). In addition, PIDs are more prevalent in areas with highly consanguineous populations due to the predominance of autosomal recessive disorders (12). The frequency of consanguineous unions exceeds 50% in many the Middle Eastern countries (13), consequently, the frequency of PIDs is believed to be greater in these countries compared to North America and Europe. Several registries were established at the regional, national, and institutional levels to document and study patients with PIDs (11, 14–26). These registries provided invaluable epidemiological and clinical information related to the different diseases and facilitated research collaborations to understand the functional and genetic basis of many disorders. Importantly, registries highlighted manifestations of PIDs other than infections (6).

The Kuwait National Primary Immunodeficiency Registry (KNPIDR) was established in 2007 (14). Several reports were published describing the demographic, epidemiologic, and both infectious and non-infectious manifestations of the patients included in the registry (14, 27–30). In this manuscript, we report the frequency and manifestations of autoimmunity in children with PIDs registered in the KNPIDR from January 2004 to December 2019.

## Materials and Methods

### Patients Data

The data was obtained from the KNPIDR, as part of a study approved by The Research and Ethics Committee of the Ministry of Health in Kuwait, and by the Kuwait University Health Sciences Center Ethical Committee, in accordance with the Declaration of Helsinki. The patients were followed prospectively, and they were categorized according to the International Union of Immunological Societies (IUIS), Primary Immunodeficiency Diseases Committee Report on Inborn Errors of Immunity (2019) (1). Secondary immunodeficiencies were ruled out by obtaining a complete history and by performing proper testing when these disorders were suspected.

### Diagnosis of Autoimmunity

The diagnosis of autoimmunity was based on the standard of care depending on the patient's signs and symptoms, supported by laboratory testing when needed. For instance, patients who were diagnosed with autoimmune hemolysis presented with anemia that was associated with a positive Coombs test.

### Statistics

Data were processed using Minitab 19.2020.1 (Minitab LLC, Pennsylvania, USA). Pearson's Chi-square test was used to assess whether autoimmune manifestations have any relation with death count or PIDs categories. The non-parametric Mann–Whitney *U*-test was applied to test whether the patients' ages at onset of a symptom of PIDs have a significant effect on the risk of developing autoimmunity. A two-sample *t*-test was used to assess whether there is a difference in diagnosis delay between the group of patients with and without autoimmunity. One-way ANOVA test was used to determine whether there is a difference in diagnosis delay in patients with autoimmune manifestation with respect to PID categories. The Kaplan–Meier survival analysis was used to estimate the survival function. Survivals were calculated from the date of diagnosis until the date of death (uncensored) or until the end of the study period (censored). The *p* ≤ 0.05 was used as the cut-off level for statistical significance.

## Results

### Patients

A total of 286 PID children (146 males and 140 females) were registered in KNPIDR during the study period. The distribution of these patients according to PID categories showed predominance of immunodeficiencies affecting cellular and humoral immunity (36%), followed by combined immunodeficiencies with associated syndromic features (23.5%) and diseases of immune dysregulation (16.4%) (Table 1). The mean and median ages at onset of PID symptoms were 13 and 3 months, respectively (0–312 months) while the mean and median ages at diagnosis were 35 and 10 months (0–456 months), respectively. The mean and median delay in diagnosis were 21 and 5 months (0–372 months), respectively.

**Table 1:** Frequency of autoimmune disorders in PID patients registered in the Kuwait National Primary Immunodeficiency Registry.

**Category**	**Total number and percentage (%) of patients**	**Number and percentage (%) of patients with autoimmunity**
Immunodeficiencies affecting cellular and humoral immunity	103 (36.0)	20 (19.4)
Combined immunodeficiencies with associated or syndromic features	67 (23.4)	8 (11.9)
Predominantly antibody deficiencies	35 (12.3)	5 (14.3)
Diseases of immune dysregulation	47 (16.4)	17 (36.2)
Congenital defects of phagocyte number or function	21 (7.3)	2 (9.5)
Autoinflammatory disorders	1 (0.4)	1 (100)
Complement deficiency	12 (4.2)	4 (33.3)
Total	286 (100)	57 (19.9)

### Characteristics of Autoimmune Manifestations

**Figure 1:**
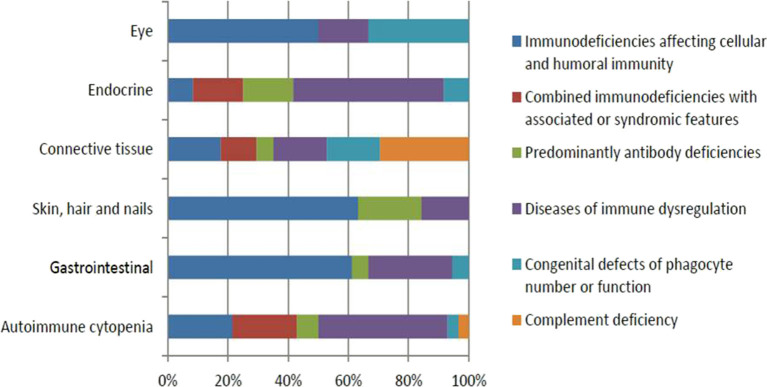
The distribution of different autoimmune manifestations with respect to PID categories.

Fifty-seven (19.9%) patients presented with a total of 107 autoimmune manifestations. Patients with autoimmune manifestations were older at onset of PID symptoms (mean age 19 months; Table 2) compared to those with no such manifestations (mean age 12 months), but this did not reach level of significance (*p* = 0.149). The diagnosis delay was longer in patients with autoimmune manifestations (31 months) compared to the group with no such manifestations (19 months) (*p* = 0.038). Also, mean times of diagnosis delay in patients with autoimmune manifestations across PID categories was found to be significantly different (*p* = 0.032). There was no statistically significant association between having autoimmunity and gender (*p* = 0.926; Table 2). Forty-seven percent of these manifestations were among the presenting symptoms at the time of PID diagnosis while 53% were documented after establishing the diagnosis. Thirty-one patients (54%) developed 1 autoimmune manifestation, 17 patients (30%) developed 2 such manifestations, and 9 (16%) patients had ≥3 autoimmune manifestations. Patients with polyautoimmunity were not different compared to those with only one autoimmune manifestation with respect to PID categories or gender. Figure 1 shows the distribution of different autoimmune manifestations with respect to PID categories. The most striking findings are that autoimmune cytopenia were more common in patients with immune dysregulation syndromes, while gastrointestinal and skin manifestations predominate in patients with immunodeficiencies affecting cellular and humoral immunity and endocrine manifestations were more common in immune dysregulation syndromes.

**Table 2:** Demographic information of patients with autoimmune disorders among PID patients registered in the Kuwait National Primary Immunodeficiency Registry.

**Category**	**Number and percentage of patients**	**Gender M/F (%)**	^*^Age at PID onset and range	^*^Age at PID diagnosis and range	^*^^¥^Delay in diagnosis
Immunodeficiencies affecting cellular and humoral immunity	20 (35%)	9/11 (45/55)	14	35	21.
			0–72	0–168	0–120
Combined immunodeficiencies with associated or syndromic features	8 (14%)	4/4 (50/50)	3	35	35
			0–12	0–120	12–55
Predominantly antibody deficiencies	5 (8.8%)	2/3 (40/60)	38	73	32
			12–48	30–101	0–120
Diseases of immune dysregulation	17 (29.9%)	11/6 (65/35)	23	54	30
			0–113	10–156	1–154
Congenital defects of phagocyte number or function	2 (3.5%)	1/1 (50/50)	48	54	6
			0–96	1–108	1–12
Autoinflammatory disorders	1 (1.8%)	1 F	6	15	
Complement deficiency	4 (7%)	2/2 (50/50)	30	134	104
			7–60	76–240	30–198
Total	57 (100%)	29/28	19	51	31
			0–113	0–240	0–198

*PID, primary immunodeficiency disease; M, male; F, female*.

* *Mean (months)*.

¥ *p-value = 0.032*.

Autoimmune manifestations were more common in patients with immune dysregulation (36.2%) followed by complement deficiencies (33.3%; Table 1). There was a statistically significant association between having autoimmune manifestations and PID category after excluding patients who belong to congenital defects of phagocyte number or function, autoinflammatory disorders, and complement deficiencies due to low numbers (*p* = 0.02; Table 1). The most common immune manifestations consisted of autoimmune cytopenia (27.1%) (Table 3). This was followed by gastrointestinal manifestations (20.6%), and manifestations of the skin, hair, and nails (20.6%). The frequency of autoimmunity was high among RAG-deficient patients, with 10 out of 18 patients (55.6%) showing at least one autoimmune manifestation. Autoimmunity was also prevalent among patients with Wiskott–Aldrich Syndrome (two out of three patients), STAT5b deficiency (two out of two patients), NF-kB2 deficiency (two out of two patients), Fas and FasL deficiency (three out of four patients), LRBA deficiency (three out of three patients), APECED (three out of three patients), IL-10 deficiency (four out of four patients), and complement C4 deficiency (four out of five patients; Table 4). There were also nine patients with autoimmunity but no identified genetic defect.

**Table 3:** Categorization of the 107 autoimmune manifestations observed in 57 patients with primary immunodeficiency disease.

**Manifestations**	**Number**	**Percentage**
Autoimmune cytopenia	29	27.1
Gastrointestinal	22	20.6
Skin, hair, and nails	22	20.6
Connective tissue	14	13.0
Endocrine	12	11.3
Eye	7	6.5
Kidney	1	0.9
Total	107	100

**Table 4:** Spectrum of autoimmune disorders among PID patients registered in the Kuwait National Primary Immunodeficiency Registry.

**Category**	**Number of patients**	**Autoimmune manifestations**
**Immunodeficiencies affecting cellular and humoral immunity**	**20**	
CD3??deficiency	2	Omenn syndrome
RAG-1 deficiency	6	Omenn syndrome, inflammatory bowel disease
RAG-2 deficiency	4	Hemolytic anemia, arthritis, psoriasis, alopecia
Artemis deficiency	1	Omenn syndrome
RFXANK-mediated MHC II deficiency	1	Hemolytic anemia, thyroiditis
ICOS deficiency	1	Enteropathy
DOCK2 deficiency	1	Anticardiolipin syndrome
DOCK8 deficiency	1	Uveitis, retinitis, chorioiriditis, vasculitis
Other combined immunodeficiencies	3	Omenn syndrome, hemolytic anemia, neutropenia, thrombocytopenia, granulomatous dermatitis
**Combined immunodeficiencies with associated or syndromic features**	**8**	
Wiskott–Aldrich syndrome	2	Hemolytic anemia, inflammatory bowel disease, thrombocytopenia, vasculitis
DiGeorge syndrome	2	Hemolytic anemia, thrombocytopenia, neutropenia,
STAT5b deficiency	2	Thyroiditis, arthritis
ICF syndrome with no documented genetic defect	2	Enteropathy
**Predominantly antibody deficiencies**	**5**	
AID deficiency	1	Systemic lupus erythematosus, thrombocytopenia
NF-?B2 deficiency	2	Anti-thyroid antibodies, alopecia, nail lichen planus, psoriasis, adrenal failure
Selective IgA deficiency	1	Celiac disease
CVID with no documented genetic defect	1	Hemolytic anemia
**Diseases of immune dysregulation**	**17**	
Chediak-Higashi syndrome	1	Thrombocytopenia
Fas deficiency	2	Anti-thyroid antibodies, vasculitis of the skin, neutropenia
FasL deficiency	1	Thrombocytopenia, hemolytic anemia
LRBA deficiency	3	Thrombocytopenia, hemolytic anemia
APECED	3	Adrenal failure, hypothyroidism, hypoparathyroidism, alopecia, autoimmune hepatitis, interstitial keratitis
IL-10 deficiency	4	Inflammatory bowel disease
Other immune dysregulation disorders	3	Hemolytic anemia, discoid lupus, enteropathy, psoriasis, vitiligo, thyroiditis, arthritis, thrombocytopenia
**Congenital defects of phagocyte number or function**	**2**	
CYBA deficiency	1	Livedo reticularis, anticardiolipin syndrome, inflammatory bowel disease
NCF2 deficiency	1	Arthralgia, hemolytic anemia, hypothyroidism, systemic lupus erythematosus
**Autoinflammatory disorders**	**1**	
Blau syndrome	1	Uveitis
**Complement deficiency**	**4**	
C4 deficiency	4	Hemolytic anemia, uveitis, arthritis, systemic lupus erythematosus, lupus nephritis, iriditis

*PID, primary immunodeficiency disease; MHC, major histocompatibility complex; ICF, immunodeficiency, centromeric instability, facial anomalies; CVID, common variable immunodeficiency; APECED, Autoimmune polyendocrinopathy-candidiasis-ectodermal dystrophy*.

### Survival Analysis

**Figure 2:**
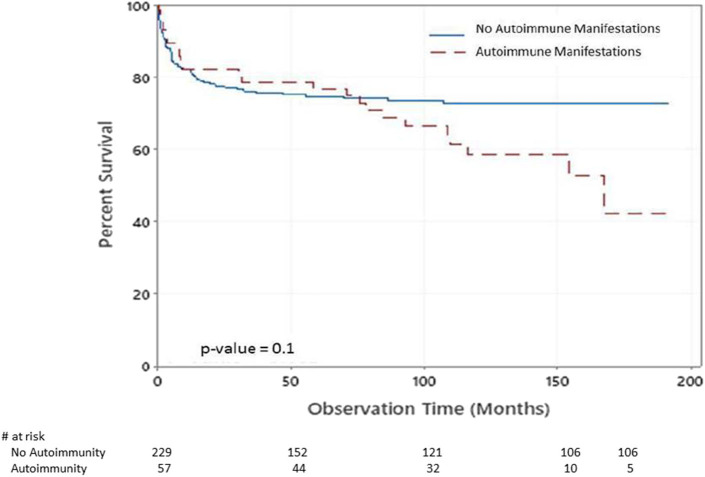
Kaplan–Meier survival plot of the chance of survival among children with PIDs with respect to having autoimmune manifestations.

There were 83 deaths during the study period. The causes of death were pneumonia, sepsis, multiorgan failure, renal failure, pulmonary hemorrhage, brain hemorrhage, liver failure, severe obstructive cardiomyopathy, and car accident. There was significant statistical association between having autoimmune manifestations and death [*p* = 0.028 (16.34% of alive patients have autoimmune manifestation compared to 27.71% of those who are dead)]. Figure 2 shows the Kaplan–Meier survival curves of children with PIDs with respect to having autoimmune manifestations. Although there was no statistical significance at α = 0.05 (*p* = 0.1), the probabilities that patients with no autoimmune manifestations survived 2, 6, and 14 years after diagnosis were 77, 74, and 72%. At the same time, they were 82, 74, and 42% among patients with autoimmune manifestations.

## Discussion

The present prospective study reports the frequency and manifestations of autoimmunity in patients with PIDs from Kuwait. The patients included in this study have an established PIDs and were reported previously (14). This patient population is unique in its homogeneity because Kuwait is a conservative country in the Arabian Peninsula where traditional consanguineous marriages are common. Therefore, genetic etiologies that lead to PIDs and autoimmunity are expected to be preserved in the gene pool of the population, and the predominance of autosomal recessive autoimmune diseases are expected to be enriched. Although autoimmunity in patients with PIDs has been extensively reported, to our knowledge, this is the first systematic report of autoimmunity in patients with PIDs from the Middle East and North Africa. What made this study possible is the establishment of the KNPIDR where autoimmunity was one of the clinical manifestations recorded for each patient with PIDs enrolled.

Prior to our study, a study that included data from 2,183 patients registered between September 1, 2013 and February 1, 2016 in the French national registry, reported that 26.2% of the PIDs patients suffered from at least 1 autoimmune manifestation, and demonstrated that autoimmune and inflammatory diseases are more frequent by a factor of 10 in patients with PIDs than in the general population (6). This French study was the first to survey autoimmunity in patients with PIDs recorded in a registry, therefore, a direct comparison of our data with the data included in the French paper is appropriate. In our population, around 20% of the 286 patients suffered from autoimmunity. The lower percentage observed in our population compared to the French study is probably due to the fewer number of patients included in our study. Another factor could be the young age of the patients, who, if followed for a longer time, then some patients might develop autoimmunity at an older age. Because of their serious clinical conditions, some of the patients from our registry were transplanted to correct their immune system, so a follow up of the possibility of developing autoimmunity at a later age due to their initial PIDs is needed. It is important to mention that ethnicity and environmental factors could have contributed to the higher frequency of autoimmunity in the French registry compared to ours. The longer delay in diagnosis in the group of patients with autoimmune manifestations compared to the group with no such manifestations is probably due to the less severe non-infectious presentations, which resulted in delay in testing for PIDs. In contrast to the French study, there was no relation between autoimmunity and the probability of survival in our cohort at a specific time interval as assessed by the Kaplan–Meier estimator. However, the occurrence of autoimmunity in our patients affected the overall outcome (alive or dead). This highlights the importance of choosing therapeutic options that are safe and non-toxic to control disease manifestations. There were considerations at one point to treat autoimmunity in patients with non-sever PIDs with stem cell transplant. However, this is becoming less favorable now due to the availability of targeted therapy with biologics and small molecules (31).

The total number of patients with autoimmunity was highest among patients with immunodeficiencies affecting cellular and humoral immunity because this category included the largest number of patients with PIDs. This category is mainly characterized by low to absent T cells, or present but poorly functional T cells (1). In our cohort of 18 RAG-deficient patients, 10 (55.6%) of them exhibited autoimmunity. This suggests that some autoreactive T and/or B cell clones developed, escaped central negative selection, and expanded in the periphery resulting in autoimmunity, which is very common in Omenn syndrome due to partial RAG or ARTEMIS deficiency (32). This may be compounded by the absence of Treg cells, or presence of dysfunctional Treg cells that leaves the autoreactive T cells unchecked (33).

The largest percentage of patients who showed clinical signs of autoimmunity (36.2%) was in the category of diseases of immune dysregulation, highlighting the role of lymphocyte homeostasis (Fas/FasL deficiency), immunosuppressive cytokines (IL-10 deficiency), and CTLA-4 surface expression (LRBA deficiency) in the control of autoimmunity. Furthermore, 75% of the patients with Wiskott-Aldrich syndrome, and 100% of the patients with STAT5b deficiency suffered from autoimmunity. This finding is not surprising due to the important role of WASp in Treg cell homeostasis (34), and that of STAT5b in transmitting IL-2 signaling, which is primordial for the survival and function of Treg cells (35).

The most commonly observed autoimmune manifestation in our cohort is autoimmune cytopenia (27.1%) that included hemolytic anemia, neutropenia, and thrombocytopenia. This finding is in accordance with several studies that reported autoimmune cytopenia as the most common autoimmune manifestation observed in patients with PIDs and may be a typical first symptom of an immunodeficiency that should trigger an immune evaluation (6, 36–38). Possible causes of cytopenia in PIDs comprise cellular or humoral autoimmunity, immune dysregulation in the form of hemophagocytosis or lymphoproliferation with or without splenic sequestration, bone marrow failure, and myelodysplasia, or secondary myelosuppression (38, 39).

Gastrointestinal manifestations and manifestations of the skin, hair, and nails were also common (20.6% each). This is comparable to the percentages found in the French registry (24.4 and 14.1%, respectively) (6). Omenn syndrome is characterized by early onset generalized erythroderma intractable diarrhea, failure to thrive, alopecia, lymphadenopathy, and hepatosplenomegaly. Hence, we included the manifestations of these patients in both gastrointestinal and skin/hair/nail manifestations.

Some limitations of our study include the relatively low number of patients included and the short follow up time. However, this study represents the initial report from our registry and should be viewed as an incentive for future studies. Other reports are likely to follow and will include a larger number of patients who will be followed longitudinally. We will also build on the current report to expand the range of clinical information obtained from the current or future patients in order to capture a better picture of the incidence of autoimmunity. Another limitation of the current report is that it did not determine the impact of autoimmune manifestations on the patients and on the health system. This would have been very beneficial and could be established by having immunodeficiency and dysregulation activity score based on severity of organ involvement and infections, days of hospitalization, supportive care requirements, and performance indices (40). It is important to highlight that collaborative efforts are highly needed to collect a bigger number of patients and to compare the characteristics of autoimmune manifestations between PID patients from different ethnicities and geographic areas.

In summary, autoimmunity is frequent in patients with PIDs in Kuwait, and autoimmunity coexists with PIDs, may be due in part to the improved care of patients with PIDs and their longer lifespan, as time is typically needed for some autoimmune phenomena to develop. When it manifests in PID patients, autoimmunity could worsen the clinical picture, shorten the survival time, and affect the treatment outcome. Furthermore, PIDs should be suspected in patients who present with autoimmunity, especially autoimmune cytopenia.

## Data Availability Statement

The raw data supporting the conclusions of this article will be made available by the authors, without undue reservation.

## Ethics Statement

The studies involving human participants were reviewed and approved by The Research and Ethics Committee of the Ministry of Health in Kuwait, and by the Kuwait University Health Sciences Center Ethical Committee, in accordance with the Declaration of Helsinki. Written informed consent from the participants' legal guardian/next of kin was not required to participate in this study in accordance with the national legislation and the institutional requirements.

## Author Contributions

MM: Participation in patients' diagnosis, data analysis, writing the manuscript, approval of the submitted manuscript, and agreement to be accountable for the content of the work. MZ: Data analysis and statistics, and approval of the submitted manuscript and agreement to be accountable for the content of the work. WA-H: Establishment and funding of the KNPIDR, patients' diagnosis, data collection and analysis, writing the manuscript, approval of the submitted manuscript, and agreement to be accountable for the content of the work.

## Conflict of Interest

The authors declare that the research was conducted in the absence of any commercial or financial relationships that could be construed as a potential conflict of interest.

## References

[1] Tangye SG Al-Herz W Bousfiha A Chatila T Cunningham-Rundles C Etzioni A Human inborn errors of immunity: 2019 update on the classification from the International Union of Immunological Societies Expert Committee. J Clin Immunol. (2020) 40:24–64. 10.1007/s10875-019-00737-x 31953710 PMC7082301

[2] Goyal R Bulua A Nikolov N Schwartzberg P Siegel R. Rheumatologic and autoimmune manifestations of primary immunodeficiency disorders. Curr Opin Rheumatol. (2009) 21:78–84. 10.1097/BOR.0b013e32831cb939 19077721 PMC2760066

[3] Kolhatkar NS Brahmandam A Thouvenel CD Becker-Herman S Jacobs HM Schwartz MA. Altered BCR and TLR signals promote enhanced positive selection of autoreactive transitional B cells in Wiskott-Aldrich syndrome. J Exp Med. (2015) 212:1663–77. 10.1084/jem.20150585 26371186 PMC4577851

[4] Magnani A Brosselin P Beaute J de Vergnes N Mouy R Debre M. Inflammatory manifestations in a single-center cohort of patients with chronic granulomatous disease. J Allergy Clin Immunol. (2014) 134:655–62. 10.1016/j.jaci.2014.04.014 24985400

[5] Hoyt K Chatila T Notarangelo L Hazen M Janssen E Henderson L. The immunologic features of patients with early-onset and polyautoimmunity. Clin Immunol. (2020) 211:108326. 10.1016/j.clim.2019.108326 31838215 PMC7050734

[6] Fischer A Provot J Jais J. Autoimmune inflammatory manifestations occur frequently in patients with primary immunodeficiencies. J. Allergy Clin. Immunol. (2017) 140:1388–393. 28192146 10.1016/j.jaci.2016.12.978

[7] Grimbacher B Warnatz K Yong P Korganow A Peter H. The crossroads of autoimmunity and immunodeficiency: lessons from polygenic traits and monogenic defects. J Allergy Clin Immunol. (2016) 37:3–17. 10.1016/j.jaci.2015.11.004 26768758

[8] Wakeland E Liu K Graham R. Delineating the genetic basis of systemic lupus erythematosus. Immunity. (2001) 15:397–408. 10.1016/S1074-7613(01)00201-1 11567630

[9] Okada Y Wu D Trynka G Raj T Terao C Ikari K. Genetics of rheumatoid arthritis contributes to biology and drug discovery. Nature. (2014) 506:376–81. 10.1038/nature12873 24390342 PMC3944098

[10] Bousfiha AA Jeddane L Ailal F Benhsaien I Mahlaoui N Casanova JL. Primary immunodeficiency diseases worldwide: more common than generally thought. J Clin Immunol. (2013) 33:1–7. 10.1007/s10875-012-9751-7 22847546

[11] Mahlaoui N Jais JP Brosselin P Mignot C Beaurain B Brito C. Prevalence of primary immunodeficiencies in France is underestimated. J Allergy Clin Immunol. (2017) 40:1731–3. 10.1016/j.jaci.2017.06.020 28732644

[12] Al-Herz W Aldhekri H Barbouche M Rezaei N. Consanguinity and primary immunodeficiencies. Hum Hered. (2014) 77:138–43. 10.1159/000357710 25060276

[13] Hadizadeh H Salehi M Khoramnejad S Vosoughi K Rezaei N. The association between parental consanguinity and primary immunodeficiency diseases: a systematic review and meta-analysis. Pediatr Allergy Immunol. (2017) 28:280–7. 10.1111/pai.12685 27893166

[14] Al-Herz W Al-Ahmad M Al-Khabaz A Husain A Sadek A Othman Y The Kuwait national primary immunodeficiency registry 2004-2018. Front Immunol. (2019) 10:1754 10.3389/fimmu.2019.01754 31396239 PMC6668014

[15] Seidel MG Kindle G Gathmann B Quinti I Buckland M van Montfrans J. The European society for immunodeficiencies (ESID) registry working definitions for the clinical diagnosis of inborn errors of immunity. J Allergy Clin Immunol Pract. (2019) 7:1763–70. 10.1016/j.jaip.2019.02.004 30776527

[16] Al-Tamemi S Naseem SU Al-Siyabi N El-Nour I Al-Rawas A Dennison D. Primary immunodeficiency diseases in Oman: 10-year experience in a tertiary care hospital. J Clin Immunol. (2016) 36:785–92. 10.1007/s10875-016-0337-7 27699572

[17] Errante PR Franco JL Espinosa-Rosales FJ Sorensen R Condino-Neto A. Advances in primary immunodeficiency diseases in Latin America: epidemiology, research, and perspectives. Ann N Y Acad Sci. (2012) 1250:62–72. 10.1111/j.1749-6632.2011.06289.x 22364447

[18] Gathmann B Goldacker S Klima M Belohradsky BH Notheis G Ehl S. The German national registry for primary immunodeficiencies (PID). Clin Exp Immunol. (2013) 173:372–80. 10.1111/cei.12105 23607573 PMC3722937

[19] Ishimura M Takada H Doi T Imai K Sasahara Y Kanegane H. Nationwide survey of patients with primary immunodeficiency diseases in Japan. J Clin Immunol. (2011) 31:968–76. 10.1007/s10875-011-9594-7 21956496

[20] Kirkpatrick P Riminton S. Primary immunodeficiency diseases in Australia and New Zealand. J Clin Immunol. (2007) 27:517–24. 10.1007/s10875-007-9105-z 17588141

[21] Stray-Pedersen A Abrahamsen TG Froland SS. Primary immunodeficiency diseases in Norway. J Clin Immunol. (2000) 20:477–85. 10.1023/A:1026416017763 11202238

[22] Mellouli F Mustapha IB Khaled MB Besbes H Ouederni M Mekki N. Report of the Tunisian Registry of primary immunodeficiencies: 25-years of experience (1988-2012). J Clin Immunol. (2015) 35:745–53. 10.1007/s10875-015-0206-9 26464197

[23] Bousfiha AA Jeddane L El Hafidi N Benajiba N Rada N El Bakkouri J. First report on the Moroccan registry of primary immunodeficiencies: 15 years of experience (1998-2012). J Clin Immunol. (2014) 34:459–68. 10.1007/s10875-014-0005-8 24619622

[24] Shillitoe B Bangs C Guzman D Gennery AR Longhurst HJ Slatter M. The United Kingdom Primary Immune Deficiency (UKPID) registry 2012 to 2017. Clin Exp Immunol. (2018) 192:284–91. 10.1111/cei.13125 29878323 PMC5980391

[25] Ehlayel M Bener A Laban M. Primary immunodeficiency diseases in children: 15 year experience in a tertiary care medical center in Qatar. J Clin Immunol. (2013) 33:317–24. 10.1007/s10875-012-9812-y 23054346

[26] Abolhassani H Kiaee F Tavakol M Chavoshzadeh Z Mahdaviani SA Momen T. Fourth update on the Iranian national registry of primary immunodeficiencies: integration of molecular diagnosis. J Clin Immunol. (2018) 38:816–32. 10.1007/s10875-018-0556-1 30302726

[27] Al-Herz W Moussa M. Survival and predictors of death among primary immunodeficient patients: a registry-based study. J Clin Immunol. (2012) 32:467–73. 10.1007/s10875-011-9636-1 22205205

[28] Al-Herz W Nanda A. Skin manifestations in primary immunodeficient children. Pediatr Dermatol. (2011) 28:494–501. 10.1111/j.1525-1470.2011.01409.x 21453308

[29] Al-Herz W Essa S. Spectrum of viral infections among primary immunodeficient children: report from a national registry. Front Immunol. (2019) 10:1231. 10.3389/fimmu.2019.01231 31191561 PMC6549382

[30] Owayed A Al-Herz W. Sinopulmonary complications in subjects with primary immunodeficiency. Respir Care. (2016) 61:1067–72. 10.4187/respcare.04479 26957648

[31] Delmonte OM Notarangelo LD. Targeted therapy with biologicals and small molecules in primary immunodeficiencies. Med Princ Pract. (2020) 29:101–12. 10.1159/000503997 31597133 PMC7098309

[32] Marrella V Maina V Villa A. Omenn syndrome does not live by V(D)J recombination alone. Curr Opin Allergy Clin Immunol. (2011) 11:525–31. 10.1097/ACI.0b013e32834c311a 22001740

[33] Cassani B Poliani P Moratto D Sobacchi C Marrella V Imperatori L. Defect of regulatory T cells in patients with Omenn syndrome. J Allergy Clin Immunol. (2010) 125:209–16. 10.1016/j.jaci.2009.10.023 20109747

[34] Humblet-Baron S Sather B Anover S Becker-Herman S Kasprowicz DJ Khim S. Wiskott-Aldrich syndrome protein is required for regulatory T cell homeostasis. J. Clin. Invest. (2007) 117:407–18. 10.1172/JCI29539 17218989 PMC1764857

[35] Cohen A Nadeau K Tu W Hwa V Dionis K Bezrodnik L. Cutting edge: decreased accumulation and regulatory function of CD4CD25(high) T cells in human STAT5b deficiency. J Immunol. (2006) 177:2770–4. 10.4049/jimmunol.177.5.2770 16920911

[36] Bussone G Mouthon L. Autoimmune manifestations in primary immune deficiencies. Autoimmun Rev. (2009) 332–6. 10.1016/j.autrev.2008.11.004 19028607

[37] Seidel M. Autoimmune and other cytopenias in primary immunodeficiencies: pathomechanisms, novel differential diagnoses, and treatment. Blood. (2014) 124:2337–44. 10.1182/blood-2014-06-583260 25163701 PMC4192747

[38] Hadjadj J Aladjidi N Fernandes H Leverger G Magérus-Chatinet A Mazerolles F. Pediatric evans syndrome is associated with a high frequency of potentially damaging variants in immune genes. Blood. (2019) 134:9–21. 10.1182/blood-2018-11-887141 30940614

[39] Notarangelo L. Primary immunodeficiencies (PIDs) presenting with cytopenias. Hematology Am Soc Hematol Educ Program. (2009) 2009:139–43. 10.1182/asheducation-2009.1.139 20008192

[40] Tesch VK Abolhassani H Shadur B Zobel J Mareika Y Sharapova S. Long-term outcome of LRBA deficiency in 76 patients after various treatment modalities as evaluated by the immune deficiency and dysregulation activity (IDDA) score. J Allergy Clin Immunol. (2019) 145:1452–63. 10.1016/j.jaci.2019.12.896 31887391

